# Body Dissatisfaction and Mirror Exposure: Evidence for a Dissociation between Self-Report and Physiological Responses in Highly Body-Dissatisfied Women

**DOI:** 10.1371/journal.pone.0122737

**Published:** 2015-04-01

**Authors:** Fátima Servián-Franco, Silvia Moreno-Domínguez, Gustavo A. Reyes del Paso

**Affiliations:** Department of Psychology, University of Jaén, Jaén, Spain; University of Tuebingen Medical School, GERMANY

## Abstract

**Background:**

Weight and shape concerns are widespread in the general population. Mirror exposure has been used to reduce body dissatisfaction but little is known about the mechanisms which underlie this therapeutic technique. The present study examined emotional, cognitive, and psychophysiological responses, in women with high and low levels of body dissatisfaction, exposed to their own bodies in a mirror.

**Method:**

Forty-two university-attending women (21 high body-dissatisfied (HBD) and 21 low body-dissatisfied (LBD)), were confronted with their own body during four 5-min trials in which participants were instructed to focus their attention on different parts of their body under standardized conditions. Emotional and cognitive measures were taken after each exposure trial. Heart rate (HR) and skin conductance (SC) were recorded continuously.

**Results:**

HBD women experienced more negative emotions and cognitions following body exposure compared to LBD women but, conversely, showed a reduced physiological reaction in terms of HR and SC. In both groups greater physiological responses were observed looking at the thighs, buttocks, and abdomen. Extent of negative emotions and cognitions were positively associated with HR and/or SC in LBD women but no associations were observed in HBD women.

**Conclusion:**

The dissociation between self-report and psychophysiological measures in HBD women supports the existence of a passive-behavioral inhibited coping style in HBD women and suggests deficiencies in the generation of physiological correlates of emotion related to body dissatisfaction.

## Introduction

Body dissatisfaction plays an important role in the development, maintenance and relapse of eating disorders (ED) [1,2], and is a central diagnostic feature of both anorexia nervosa (AN) and bulimia nervosa (BN) [3]. Dissatisfaction with one’s weight, shape, and overall appearance is increasingly prevalent in the general female population [4,5] and can be considered as a major psychological problem, responsible for a great deal of suffering in many women in modern societies [6–8]. Therefore research is also required pertaining to women who reject their own bodies but do not develop clinically-relevant ED symptoms.

Emotional functioning may be a key factor in the genesis of body dissatisfaction and the development of clinically significant ED symptoms [9,10]. Research on the role of emotional factors in the development of ED indicates that feelings of depression and negative affect may modulate, and even be directly responsible for, disordered eating and BN symptoms [11,12]. Similarly, it has also been reported that emotional disturbances, such as a high negative affect and lower levels of emotional expression, are associated with greater levels of body dissatisfaction in healthy university-attending women [13] and teenagers [14]. In females, body mass index (BMI) is a powerful predictor of body dissatisfaction and social anxiety [15].

Mirror exposure techniques (e.g. asking patients to observe themselves repeatedly and for prolonged periods in a full-length mirror) have previously been used to reduce body dissatisfaction. It is assumed that the evoked negative emotions and cognitions habituate and change during these prolonged and repeated mirror exposures [16,17]. Although previous literature generally supports the effectiveness of mirror exposure [18–20], little is known about its underlying therapeutic mechanisms. Body image disturbances may be characterized as cognitive-emotional alterations [6,21]. Cognitive models of ED propose that patients selectively attend to appearance cues [21–23] and demonstrate attentional bias in the processing of appearance-related information [23]. A relevant component of body image disturbance refers to the emotions experienced in response to the sight of one’s own body. In this context, body dissatisfaction can be considered as a set of extreme reactions encompassing disrespect, rejection and avoidance of one's own body, even when assessment of size is accurate [18,23].

Few studies have thus far been devoted to measuring psychophysiological responses that occur during mirror exposure therapy. Whether the activation of negative emotions and cognitions during body exposure are accompanied by physiological activation remains unclear. It might be that the evocation of negative emotions (e.g. fear, anger, anxiety, sadness, etc.) during exposure elicits autonomic and endocrine responses [24]. Vocks et al. [17] examined whether patients with ED exhibited greater psychophysiological responses, in addition to stronger emotional-cognitive reactions, when exposed to their own bodies in front of a mirror in comparison to control participants. They reported that ED patients exhibited higher levels of negative emotions and cognitions in response to body exposure compared to control participants, but no group differences were found for heart rate (HR), skin conductance (SC) or saliva cortisol. During body exposure, negative emotions and cognitions decreased significantly but all physiological parameters remained stable. In line with previous literature [25,26] the authors concluded that the greater emotional and cognitive reactions to body exposure in ED patients occur in the context of a reduced response capability of the sympathetic nervous system and hypothalamic-pituitary-adrenal axis, which might also explain the absence of differences at the physiological level. It is assumed that starvation in AN, and the intermittent dieting seen in BN, predispose individuals to reduced autonomic and neuroendocrine responses to stress [25,26].

To our knowledge, no research is available concerning the physiological reactions of women with high vs. low body dissatisfaction, free from ED symptomatology, when confronted with their own bodies in a mirror. Tuschen-Caffier et al. [18] reported that exposure to body shape is stressful not only for patients with BN but also for women without ED. In both cases women reacted with increased tension, anxiety, insecurity and sadness. In this context it is relevant to ask whether the stronger emotions and negative cognitions in women suffering from body image disturbances are also accompanied by stronger psychophysiological reactions.

In the present study we aimed to examine emotional, cognitive, and psychophysiological responses in women with high (but without ED symptoms) vs. low levels of body dissatisfaction when exposed to their own body in a mirror. Psychophysiological measures included HR and SC as indicators of autonomic nervous system activity. These two measures have traditionally been used to index psychological processes including emotions [27]. Greater autonomic arousal, as indexed by these measures, appears to relate to greater success in exposure-based desensitization techniques [18,29]. In accordance with Vocks et al. [17], we expected highly body-dissatisfied (HBD) women to react with larger increases in negative emotions and cognitions, in response to seeing their body image in a mirror, in comparison to low body-dissatisfied (LBD) women. Contrary to Vocks´s findings, and where our participants are neither in a state of starvation nor dieting, we also hypothesize stronger psychophysiological reactions to their body image in HBD vs. LBD women. We will also analyze associations between self-report measures and psychophysiological responses, especially with respect to possible group differences, as this may indicate alterations in affective-emotional regulation related to the appraisal of their own body in HBD women [6,21].

## Method

The experimental protocol was approved by the Bioethics Committee of the University of Jaén. Upon arrival at the laboratory, participants received detailed information about the procedure and signed an informed consent form.

### Participants

Forty-two women from the University of Jaén were recruited through advertisements, placed throughout the campus, which requested the participation of women with or without concerns about their body image. Respondents were interviewed (regarding exclusion criteria), asked to complete the Body Shape Questionnaire (BSQ) [30], and were then weighed and measured. Exclusion criteria were: a) a Body Mass Index (BMI) below 18 or above 28; b) binging or purging behaviours; c) presently being on a weight loss program; and e) undergoing concurrent psychotherapeutic treatment or frequent drug or alcohol use. Group selection was dependent on BSQ score. Participants with BSQ scores below 70 were considered LBD (*n* = 21; *M* = 52.15, *SD* = 9.21, range: 41–70) and participants with BSQ scores above 110 were considered HBD (*n* = 21; *M* = 136.33, *SD* = 17.13, range: 113–170). The groups did not differ in age (HBD: *M* = 21.33, SD = 1.31; LBD: *M* = 20.85, *SD* = 1.08) but they did differ in BMI (*t*(40) = 6.14; *p* < .01). As expected, BMI was higher in the HBD group (*M* = 24.46, *SD* = 2.49) in comparison to the LBD group (*M* = 20.62, *SD* = 1.73). Participants received 20€ for their participation. One LBD participant was excluded from the SC measure owing to equipment problems (thus rendering a LBD group *n* of 20 for the SC measure).

### Self-report measures

Trait body dissatisfaction was measured via the BSQ [30], a 34-item questionnaire assessing body image concerns, especially with respect to feelings of fatness (and where a low BSQ score should not necessarily be interpreted as indicative of body satisfaction). Each item is rated using a scale from *Never* (1) to *Always* (6). Scores range from 34 to 204; the clinical cut-off point is 105 [30]. The BSQ has been validated in Spanish populations and exhibits good reliability and validity [31]. In our study the Cronbach´s α was. 98.

State body dissatisfaction and mood were assessed using visual analogue scales (VASs). Participants responded to the question: “How do you feel right now” by placing a mark on a 10 cm line anchored by “*not at all”* (at 0 cm) on the left and *“completely*” (at 10 cm) on the right. To assess body image, the words *Body discomfort* and *Ugly* were employed. To assess mood, participants responded to the words *Shame*, *Sadness*, *Anger*, *Fear*, *Insecure* and *Tension*. Heinberg and Thomson [32] demonstrated that VAS are reliable indices of changes in mood and extent of body dissatisfaction.

Negative thoughts were assessed by the *Thoughts Checklist* (TCL) [33]. This questionnaire consists of statements representing the typical thoughts which patients with ED have while carrying out each of the three behavioural tasks referred to in the questionnaire: the mirror, weighing and eating tasks. In the present study only the 15 items pertaining to the mirror task were employed (e. g. “*I can´t look at myself in the mirro*r” and “*my shape is out of proportion*”), rated by the frequency of their occurrence (1 = *thought did not occur;* 6 = *thought was there all the time*). The mean value for negative thoughts was computed (range: 15–90). This scale has demonstrable reliability and validity: in our study the Cronbach´s α was. 86.

### Psychophysiological measures

An electrocardiogram (ECG) was continuously recorded throughout the experiment, at a sampling rate of 1000 Hz, via a Biopac MP150 system (Biopac Systems Inc., USA). Electrodes (Ag/AgCl) were placed according to Einthoven's II derivation. The ECG raw signal was processed using the software AcqKnowledge 3.9.0, which allows for R-spike detection and quantification of HR values (in bpm). When present, artifacts in beat-by-beat HR data were corrected and edited using linear interpolation.

Skin conductance (SC) was recorded from two standard Ag/AgCl electrodes filled with isotonic electrolyte paste (0.29 g NaCl per 100 ml water) placed on the second phalanges of the third and forth finger of the left hand. The signal (in *μ*mhos) was recorded at a sample rate of 250 Hz via the Biopac MP150. An additional channel was used to record time marks associated with different body parts, thereby allowing for the synchronization of the mirror task with the physiological recoding. HR and SC averages for the five experimental periods, and response amplitude associated with the different body parts during the four exposition trials, were obtained.

### Procedure

Participants were given beige-colored light underwear to wear during the study. The experimental session comprised the following sequence: (a) 10-min resting period in which the last 5-min were taken as the baseline. During this period participants were standing (during the first 4-min they were allowed to support themselves via a stool previously adapted to their height) and covered with a dressing layer; (b) four 5-min trials, in which participants were instructed to remove the dressing layer, stand at a distance of two feet in front of the mirror and focus their attention on different parts of their bodies. Participants received specific instructions concerning where to look, via audio tape which described the following sequence: (1) open the mirror, (2) look directly ahead at the whole body, (3) the back of the body, (4) the face and head, (5) the neck and shoulders, (6) the arms, (7) the chest, (8) the abdomen, (9) the buttocks, (10) the thighs, (11) the hips, and (12) the calves. They were instructed to look at each body part for 10 s. The time interval between each audio tape instruction was 25 s. At the beginning of each instruction a time mark was sent to the physiological recording device to allow for analysis of responses pertaining to each body part. At the end of the trial the mirror was closed, the participant covered their body with the dressing layer and (seated in the stool) provided answers to all of the state measures (which took approximately 5-min). The experiment was performed in a room maintained at a temperature of approximately 25° centigrade. Participants were instructed to refrain from smoking, caffeine, alcohol, and performance of vigorous exercise in the 2 h prior to the experiment.

### Statistical analysis

VAS scores for *Shame*, *Tension*, *Insecurity*, *Anger and Fear* were highly correlated (all rs > .5, all ps < .01). In order to reduce the probability of type I errors scores were aggregated into a single *Negative Affect* score. VAS scores for *Sadness* had correlation coefficients of less than. 4 with the other scales and when aggregated within the overall *Negative Affect* score the Cronbach´s α decreased from. 57 to. 36 in the LBD. Then *Sadness* was maintained as a separate score. Cronbach´s α was. 83 for the whole sample in the final aggregated *Negative Affect* score. Self-report variables were analyzed via a 2(x5) repeated measures ANOVA, for which Group was the between-subjects factor and Period (baseline and the four trials) was the within-subjects factor.

HR and SC were analyzed in two steps. Firstly, and because we were interested in overall group differences in anticipation (baseline) and visualization (the four trials) of participants’ own bodies, average values pertaining to these measures were analyzed via the afore-described 2(x5) repeated measures ANOVA. Secondly, in order to explore phasic HR and SC responses to the visualization of the different body parts, data were analyzed via 2(x4x12) ANOVAs, with two between-subjects factors: the (four) trials and the (12) body parts across which the exposure periods were spread. In order to control for possible baseline group differences, average baseline HR / SC was included as a covariate. Physiological data were also analyzed based on differential scores with respect to the baseline prior to the opening of the mirror (peak value for each body part—pre-trial baseline). The results of this analysis yield similar results to those presented here. In order to provide information pertaining to the very high physiological values obtained in our task we chose to report absolute peak values. Visual inspection of the recordings revealed greater psychophysiological activation during the task, without any clear evidence of recovery between body-observation instructions. Under such conditions obtaining valid baseline values for responses associated with each body part is problematic: use of the habitual response amplitude parameter (peak response value—previous baseline value) tends to underestimate the real physiological responses. In order to avoid this bias we took the peak physiological value during the time window corresponding to the viewing of each body part as dependent variable. F values associated with the Wilks' lambda multivariate test statistic are provided. This method is free of sphericity assumptions and thus more suitable for repeated measures designs. Significant results and interactions were further analyzed via *t*-test comparisons for independent samples. Effect sizes were presented with the squared adjusted eta (η^2^) parameter.

Associations between self-report and physiological variables were also assessed in two steps. Firstly, exploratory Pearson correlations were calculated separately for each group. Secondly, associations for which a significant correlation was obtained were subjected to multiple regression analysis. The physiological variable (HR or SC) was taken as the dependent variable, and the self-report measure as the predictive factor. In each regression analysis we introduced the individual predictor, the group factor (i.e. as a dummy variable), and the interaction group x predictor term. A significant effect of the interaction term would be indicative of a significant group difference in the reported association. The significance level was set at p < .05.

## Results

### Self-report measures

The HBD group exhibited greater levels of *Body Discomfort* (*F*(1, 40) = 10.14, p < .0001, η^2^ = .504) and higher *Ugly* scores (*F*(1, 40) = 9.83, p < .0001, η^2^ = .496) compared to the LBD group. Significant G*roup x Period* interactions for *Body Discomfort* (*F*(4, 37) = 8.51, p < .0001, η^2^ = .460), and *Ugly* (*F*(4, 37) = 6.05, p *<*. 001, η^2^ = .377) were also found. Whereas feelings of *Body Discomfort* increased in trials (from baseline) in the HBD group (*F*(4, 17) = 9.48, p < .0001, η^2^ = .666), no change was observed in the LBD group (p = .367). Similarly, feelings of ugliness increased from baseline in the HBD group (*F*(4, 17) = 9.66, *p <*. *0001*, *η* ^*2*^ *=* .670), but no change was observed in the LBD group (p = .209; Table 1).

**Table 1:** Mean (and standard deviation) of Body Dissatisfaction (BD), Ugly, Sadness, and the aggregated Negative Affect (NA) VAS scores and the negative Thoughts Checklist (TCL) scores for the low body dissatisfaction (LBD) and high body dissatisfaction (HBD) groups.

**VAS**	**Group**	**Baseline**	**Trial 1**	**Trial 2**	**Trial 3**	**Trial 4**
**BD**	LBD	.81(.74)	1.18 (1.15)	.98 (1.40)	.85 (1.3)	.61(.62)
	HBD	2.85(2.76)+	7.02(2.43)*	6.70(2.63)*	7.06(2.49)*	6.91(2.87)*
**Ugly**	LBD	1.45(1.87)	1.87(1.89)	1.50(1.98)	1.35(2.04)	1.34(1.99)
	HBD	5.82(2.75)*	7.50(2.15)*	7.62(2.12)*	7.32(2.40)*	7.59(2.25)*
**Sadness**	LBD	1.27(1.83)	1.53(1.60)	.88(1.34)	.88(1.33)	.97(1.46)
	HBD	2.79(2.31)+	6.55(2.51)*	6.87(2.19)*	7.26(2.27)*	7.35(2.18)*
**NA**	LBD	1.45(.82)	1.451(1.21)	.86(.81)	.85(.87)	.70(.82)
	HBD	3.53(2.71)*	5.33(2.14)*	5.23(2.27)*	5.30(2.14)*	5.23(2.27)*
**TCL**	LBD	1.21(.15)	1.24(.19)	1.25(.19)	1.21(.17)	1.24(.19)
	HBD	2.88(.72)*	3.35(.85)*	3.29(.92)*	3.26(.88)*	3.36(1.01)*

Results of the *t*-tests for group comparisons within each period were also included (+ p < .05, * p < .01).

Regarding mood, the HBD group had higher aggregated *Negative Affect* (*F*(1, 40) *=* 60.40, p <.0001, η^2^ = .602) and *Sadness* scores (*F*(1, 40) = 48.47, p < .0001, η^2^ *=* .548; Table 1). Significant Group x Period interactions were also found for *Negative Affect* (*F*(4, 37) *=* 8.72, p <.0001, η^2^ = .485) and *Sadness* (*F*(4, 37) *=* 11.87, p < .0001, η^2^ = .562) scores. Scores for *Negative Affect* increased, between baseline and trials, in the HBD group (*F*(4, 17) = 6.67, p = .002, η^2^ = .611) and decreased in the LBD group (*F* (4, 17) = 9.36, p < .0001, η^2^ *=* .688). Feelings of *Sadness* increased in the HBD group (*F*(4, 17) = 6.67, p = .002, η ^2^ = .611) and decreased in the LBD group (*F*(4, 17) = 9.36, p <.0001, η ^2^ = .688; Table 1).

The HBD group had more *Negative Thoughts* compared to the LBD group (*F*(1, 40) = 5.07, p = 002, η^2^ = .337; Table 1). The Group x Period interaction was also significant (*F*(4, 37) = 4.54, p = .004, η^2^ = .312). *Negative thoughts* increased throughout trials in the HBD group (*F*(4, 17) = 5.38, p = .005, η^2^ = .531) while no change was observed in the LBD group (p = .748).

### Averaged psychophysiological measures

The HBD group exhibited decreased HR in comparison with the LBD group (*F*(1, 40) = 4.92, p = .032, η^2^ = .112; Table 2). No further effects were found. This group difference was significant during the first [*t*(40) = 2.02, p = .05], second [*t*(40) = 2.17, p = .035], third [*t*(40) = 2.46, p = .018] and fourth [*t* (40) = 2.57, p = .014] trials, but not during baseline [*t*(40) = 1.53, p = .132].

**Table 2:** Mean (and standard deviation) of averaged heart rate (HR) and skin conductance (SC) during the five experimental periods in the low body dissatisfaction (LBD) and high body dissatisfaction (HBD) groups.

	**Group**	**Baseline**	**Trial 1**	**Trial 2**	**Trial 3**	**Trial 4**
**HR (bpm)**	LBD	98.24(10.67)	99.71(8.39)	99.36(8.04)	99.38 (8.27)	99.62(8.11)
	HBD	92.32(13.74)	92.75(13.26)+	91.60(13.93)+	90.99(12.89)+	91.05(12.61)+
**SC (*μ*mhos)**	LBD	5.36(2.34)	5.29(2.35)	4.52(2.26)	4.17(2.16)	3.92(2.59)
	HBD	3.67(1.38)*	3.86(1.39)+	3.82(1.52)	3.80(1.65)	3.59(1.87)

Results of the *t*-tests for group comparisons within each period were also included (+ p < .05, * p < .01).

SC changed as a function of group (Group x Trials interaction: *F*(1,36) = 3.07, p = .028, η^2^ = .254; Table 2). No change was observed in the HBD group (*F*(4, 17) = 1.98, p = .143, η^2^ = .318), while SC decreases were seen throughout trials in the LBD group (*F*(4, 16) = 3.58, p = .029, η^2^ = .472). The HBD group exhibited decreased SC levels, in comparison with the LBD group, during baseline [*t*(39) = 2.83, *p* = .007] and the first trial [*t*(39) = 2.39, p = .022].

### Psychophysiological responses to visualization of the different body parts

**Fig 1:**
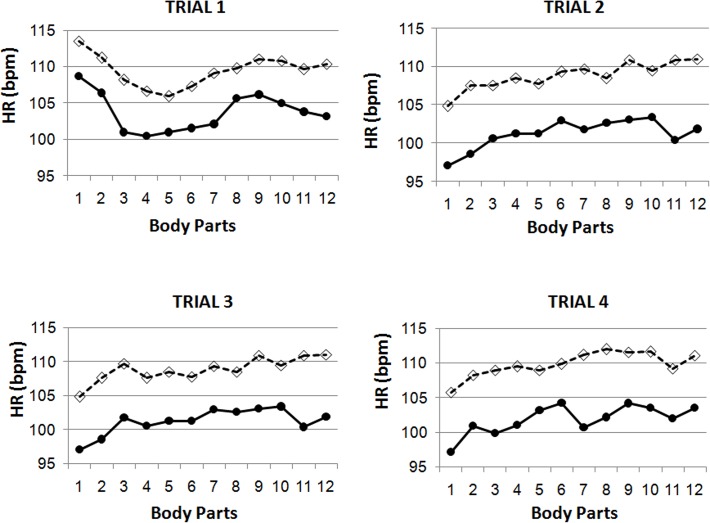
Heart rate (HR) responses. Peak HR values along the view of the 12 body parts (in the order: (1) open the mirror, (2) look ahead at the whole body, (3) back of the body, (4) face and head, (5) neck and shoulders, (6) arms, (7) chest, (8) abdomen, (9) buttocks, (10) thighs, (11) hips, and (12) calves) during the four exposure trials as a function of group. (discontinuous lines and open rhombs represent the low body dissatisfaction group and continuous lines and closed circles the high body dissatisfaction group).

HR peak values varied with the body area observed (main effect of Body Part: *F*(11,30) = 6.33, p < .0001, η^2^ = .699), and as a function of trial (Body Part x Trials interaction: *F*(33,8) = 3.24, p = .043, η^2^ = .930). As depicted in Fig 1, the response pattern during the first trial is characterized by a decrease in HR between the opening of the mirror and the viewing the neck and shoulders, a posterior increase during the viewing of the abdominal area, buttocks and thighs, and a slight decrease during the viewing of the final two body parts. For the remaining trials the response pattern is characterized by a progressive increase in HR, peaking with the viewing of the thighs, with a slight recovery during the viewing of the final two body parts.

The above effects were modulated by group (Body Part x Trials x Group interaction: *F*(33, 8) = 3.88, p = .025, η^2^ = .941). When baseline HR was introduced as a covariate the significance of this triple interaction was additionally increased (*F*(33, 7) = 5.89, p = .010, η^2^ = .965). In explaining this interaction effect, Body Part was significant in all trials in the HBD group (trial 1: *F*(11,10) = 5.91, p = .004, η^2^ = .867; trial 2: *F*(11,10) = 5.89, p = .005, η^2^ = .866; trial 3: *F*(11,10) = 6.27, *p =* .*004*, *η* ^*2*^ = .873; and trial 4: *F*(11,10) = 2.82, p = .05, η^2^ = .756). In the LBD group Body Part was significant only during the first (*F*(11,10) = 3.67, *p =* .*025*, *η* ^*2*^ = .802) and third trials (*F*(11,10) = 3.40, p = .032, η^2^ = .789).

**Fig 2:**
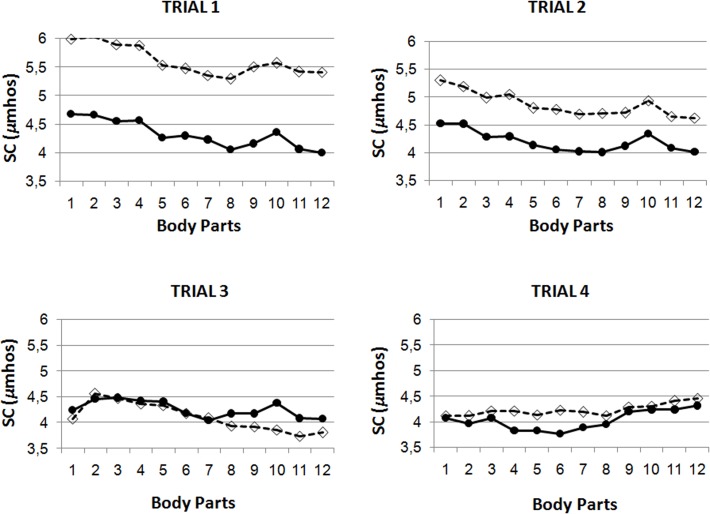
Skin conductance (SC) responses. Peak SC values along the view of the 12 body parts during the four exposure trials as a function of group. (same body parts and symbols as in Fig 1).

SC peak responses depend on the body area observed (main effect of Body Part: *F*(11, 29) = 4.69, p < .0001, η^2^ = .640). As depicted in Fig 2, the response pattern is characterized by a progressive decrease in SC across the trials, with a posterior increase during the viewing of the thighs. When mean baseline SC was introduced as a covariate, the effect Body Part is dependent on Group (Body Part x Group interaction: *F*(11, 28) = 2.14, *p =* .*005*, *η* ^*2*^ = .456). To analyze this interaction we aggregated responses to the different body parts for the four trials and analyzed them as a function of Group (using mean baseline SC as a covariate). This analysis revealed greater peak responses in the LBD group, in comparison with the HBD group, during the instruction “look ahead at the whole body” (p = .020) and marginally significant group differences for “open the mirror” (p = .085), “face and head” (p = .090), “neck and arms” (p = .075) and “back side” (p = .089).

### Associations between self-report and physiological variables

**Fig 3:**
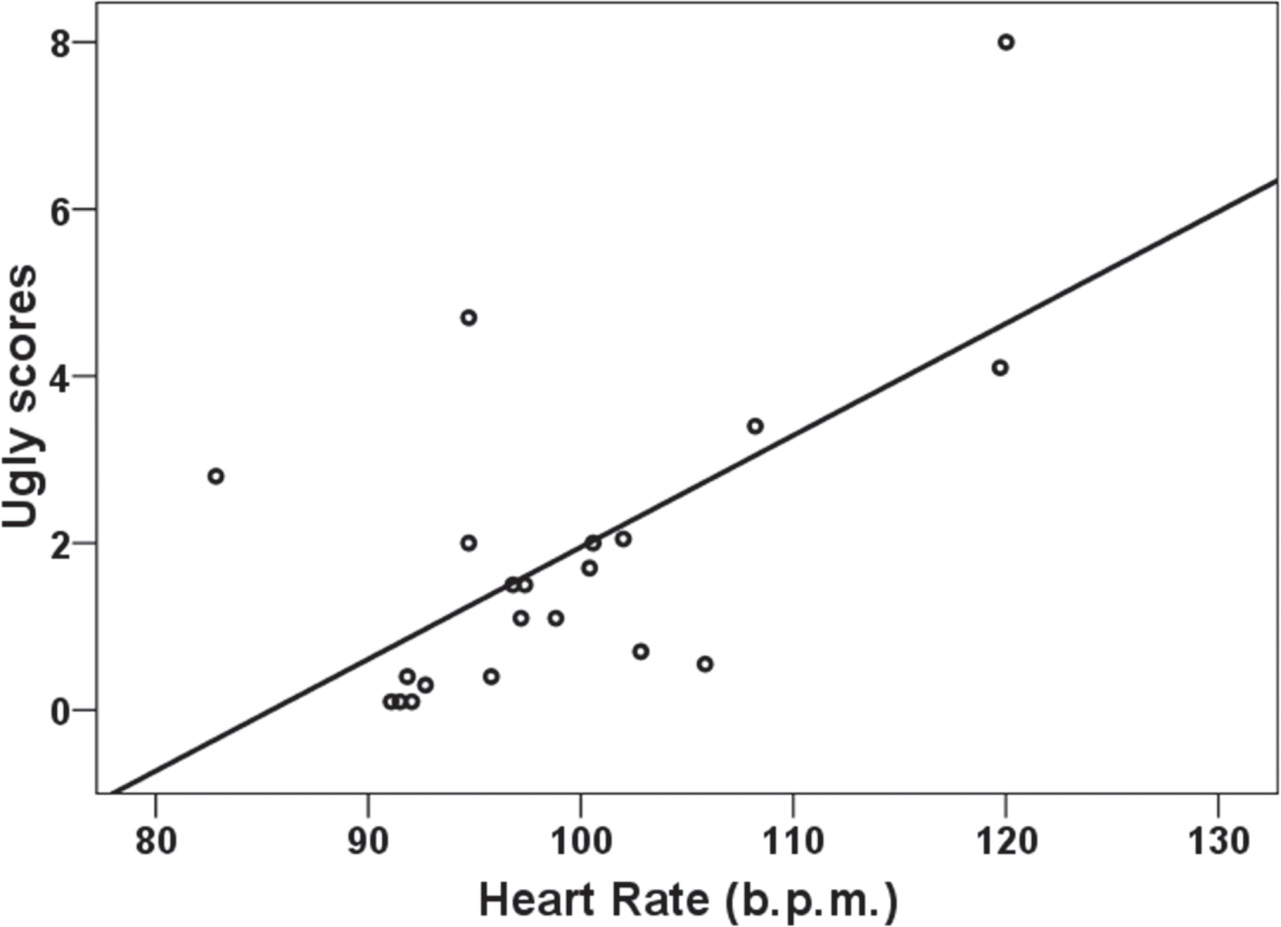
Ugly scores and heart rate (HR). Scatterplot and regression line for the relationship between ugly scores and averaged HR in the low body dissatisfaction group during exposition trial 1.

Results of the exploratory correlation analysis (averaged mean scores), for associations in which significant correlations were obtained, are displayed in Table 3. In the LBD group VAS scores for *Ugly* (in all 5 experimental periods), and *Negative Thoughts* (only during baseline), were positively associated with HR. However, these correlations were absent in the HBD group, in which a trend towards negative correlations was observed. For illustrative purposes, a scatterplot of the association between *Ugly* scores and HR is presented in Fig 3. We also analyzed correlations between *Ugly* scores and peak responses to the viewing of the different body parts. High correlations were obtained between the *Ugly* scores and responses in the LBD group, where for trials 2 and 3 all responses (except in response to the second instruction) were significantly associated with the corresponding *Ugly* scores during that trial (with rs ranging from. 436 to. 666). During trial 1 correlations only achieved significance during the viewing of the abdomen (r = .450, p = .041), buttocks (r = .515, p = .017), thighs (r = .649, p = .001) and hips (r = .458, p = .037). During trial 4 correlations only reached significance during the opening of the mirror (r = .435, p = .049). No correlation reached significance in the HBD group.

**Table 3:** Correlations between Ugly-negative Thoughts Checklist (TCL) scores and averaged heart rate (HR) and between Sadness scores and averaged skin conductance (SC).

	Group	Baseline	Trial 1	Trial 2	Trail 3	Trail 4
Ugly x HR	LBD	.564*	.624*	.662*	.663*	.499+
	HBD	-.372	-.348	-.342	-.300	.176
TCL x HR	LBD	.445+	.025	-.213	-.220	-.124
	HBD	-.245	.186	.164	.339	.310
Sadness x SC	LBD	.308	.529+	.627*	.617*	.311
	HBD	-.010	.257	.114	.315	.353

Correlations were calculated separately for the low body dissatisfaction (LBD) and high body dissatisfaction (HBD) groups.

*p <.01

+p <.05

Results of the multiple regression analysis aiming to predict HR are in accordance with the above exploratory correlational analysis. Significant *Ugly* x Group (*β* = -.331, *t* = -2.09, p = .043; *β* = -.343, *t* = -2.57, p = .014; *β* = -.349, *t* = -2.51, p = .017; *β* = -.353, *t* = -2.55, p = .015; for baseline and exposure periods 1, 2, and 3), and *Negative Thoughts* x Group (*β* = -.2.71, *t* = -2.76, p = .009; at baseline) interactions were observed, demonstrating that the obtained associations are significantly different for the two groups. While these variables are positively correlated in the LBD group, the tendency in the HBD group is towards a negative association.

**Fig 4:**
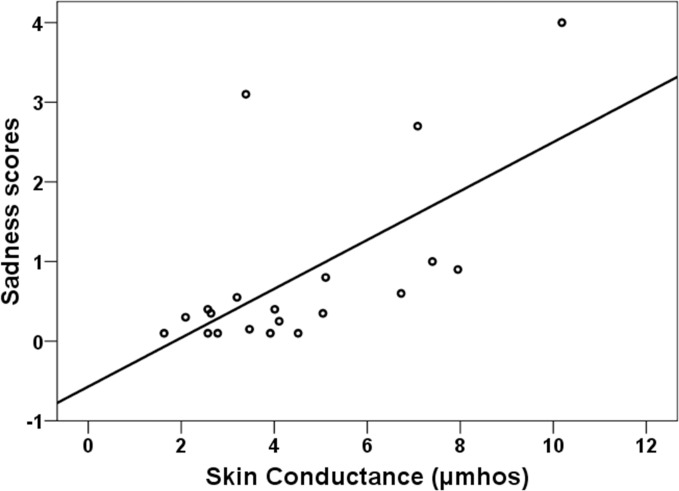
Sadness and skin conductance (SC). Scatterplot and regression line for the relationship between sadness scores and averaged SC in the low body dissatisfaction group during exposition trial 2.

The exploratory analysis also demonstrated a positive correlation between *Sadness* scores and average SC levels (Table 3), but only in the LBD group. For illustrative purposes, a scatterplot of the association between Sadness scores and SC is presented in Fig 4. We also analyzed correlations between *Sadness* scores and SC peak responses to the viewing of the different body parts. Strong correlations were observed during the two first trials in the LBD group, where all correlations were significant and ranged between. 532 and. 650. For the last two trials correlations were weaker, ranging between. 312 and. 480 in trial 3, and between. 275 and. 379 in trial 4. No correlation reached significance in the HBD group. In accordance with this association, the multiple regression analysis aiming to predict SC revealed a significant *Sadness* x Group interaction during the first three exposition periods (*β* = -.307, *t* = -2.08, p = .045; *β* = -.375, *t* = -2.42, p = .021; and *β* = -.322, *t* = -2.04, p = .048, for periods 1, 2, and 3, respectively). These results demonstrate that associations between *Sadness* scores and SC are significantly different in the two groups. The association is more positive in the LBD group compared with the HBD group.

## Discussion

The results of the present study demonstrate that HBD women (free of ED symptoms) experience more negative emotions and cognitions when confronted with their own body in a mirror than do LBD women. HBD women also scored higher in all emotional and cognitive measures at baseline. These differences replicate the results of earlier studies of ED patients [17,18,33] and HBD women free of ED symptomatology [34]. It has been argued that negative self-schemas concerning one´s own body are activated in patients suffering from ED when looking at their bodies in a mirror, thereby evoking negative emotions and cognitions [18,19,35]. The same interpretation can be applied to women dissatisfied with their bodies but who do not exhibit ED symptomatology.

Results concerning the psychophysiological measures are contrary to our hypothesis. As predicted for the emotional and cognitive measures, we also hypothesized greater HR and SC responses in the HBD group when exposed to their bodies in comparison to the LBD group. Conversely, however, the HBD group exhibited reduced autonomic arousal in the mirror task as compared to the LBD group. HBD women exhibited reduced HR during all four exposure trials, and a reduced SC level during baseline and the first trial in comparison with the LBD group. In fact, no change was found in SC across trials in the HBD group, while a habituation process was observed in the LBD group during the first two experimental periods. The high level of physiological activation observed for both measures and groups (and especially for the LBD group; e.g., averaged HR values of nearly 100 bpm), represents a remarkable finding which clearly demonstrates the stressful nature of the mirror-view task and the experimental procedure used. Furthermore, this high level of physiological activation was also observed during baseline. In view of the obtained results, this experimental phase cannot be considered as a real baseline but rather as an anticipatory phase. When dressed in light clothes, and remaining in front of the closed mirror, participants can anticipate the stressful nature of the task. Furthermore, in explaining these high physiological levels it is necessary to take into account the fact that recordings were taken while participants were standing.

Regarding specific responses to the different body parts, for HR no group difference in peak response was obtained, although the reliability of the responses appears greater in the HBD group compared with the LBD group. SC responses are greater in the LBD group compared with the HBD group for certain body parts. Body parts evoking greater responses were identical across groups. In terms of HR the body parts in question are the abdomen, buttocks and thighs. In SC a clearly identifiable peak response is observed specifically when participants of both groups are looking at the thighs. These results are in accordance with the concerns of many women, and also accord with the body parts in which fat most often accumulates in women. It is possible that responses obtained during the viewing of the thighs, even in LBD women, may relate to cellulitis concerns. Different response patterns among trials (particularly in HR) may be explained by the fact that, during trial 1, participants are naïve to the body part sequence, but not during the other trials in which they can therefore anticipate the body parts being exposed.

Previous studies have failed to obtain differences in physiological responses between ED patients and healthy controls during the mirror task. This has been explained by assuming that starvation in AN and intermittent dieting in BP affects physiological functioning by reducing autonomic and neuroendocrine reactivity [25,26]. However, in our study the reduced physiological reactivity observed in the HBD group cannot be explained by starvation nor by dieting/bingeing (these behaviors were evaluated and used as exclusionary criteria). Furthermore, we have observed a clear dissociation in the HBD group: while its emotional and cognitive responses are greater than those observed in the LBD group, its physiological reactivity was lower.

Different reasons may be offered in order to explain this dissociation. One is related to the fact that women with body image concerns spend more time looking at their body than do body-satisfied women [36]. In fact, body checking is strongly associated with body dissatisfaction and has been used to assess the effectiveness of techniques aimed at reducing body dissatisfaction [37,38]. It could be argued that the frequent practice of body checking in HBD women renders greater habituation to the body image viewing therefore resulting in an attenuated physiological reaction when confronted with their body in an experimental setting. Conversely, LBD women probably spend less time body-checking and therefore, due to the greater novelty of the situation, they might have experienced a stronger reaction when exposed to their body image in the mirror, manifesting in greater physiological reactivity. However, this cannot satisfactorily explain the heightened emotional-cognitive responses observed in the HBD group (i.e. the habituation process would have to affect both physiological and emotional responses) nor the co-occurrence of reduced emotional-cognitive responses and greater physiological reactivity in the LBD group. Body-checking behaviors have not been measured in the present study and further research is needed to clarify their impact.

An alternative, and more plausible, explanation may be that body-dissatisfied women have developed a passive coping style when confronted with their body image. Traditional psychophysiological research has distinguished between active vs. passive coping. Active coping refers to the exertion of actions to achieve control over the situation [39]. Related to perceptions of self-efficacy [40], such a style would be associated with physiological activation (e.g. increased HR) so as to allow for additional energy resources to actively interact and assist in counteracting stress [41]. Passive coping refer to a mechanisms in which an individual has no intention of actively interacting with the environment or cannot exert any control over the situation [41]. It also refers to reactions of behavioral inhibition when the individual is faced with stressful situations. As activity is not initiated or reduced, energetic mobilization is lower [39,41]. Self-rated emotional-cognitive measures in our HBD group (greater discomfort, sadness, negative affectivity, negative thoughts, etc.) suggest a state of frustration or helplessness in these women in relation to their bodies. A negative appraisal of one’s physical appearance is accompanied by discontent and a set of negative emotional reactions. Therefore, being confronted with one’s own body in a mirror represents a threatening situation for body-dissatisfied women. We predicted that, when confronted with their bodies, HBD women would react with negative emotions and feelings of frustration, because she feels that she cannot exert effective control over her body. Frustration has been related to passive coping and behavioral inhibition [42]. A lack of resources to cope with the rejection of one’s own self-image, and the consequent initiation of feelings of helplessness, may be responsible for the development of a passive coping style. The presence of a passive, behaviorally-inhibited coping style in HBD women would explain both their reduced physiological responses and greater psychological distress.

Traditional psychophysiological interpretations of HR and SC support this view. HR has been interpreted as an indicator of emotional valence, with increased HR indicative of positive emotions [27] or behavioral activation-approach [43–45] and decreased HR indicative of an unpleasant emotional experience [45]. SC has been considered an indicator of the arousal dimension of emotion [27] and behavioral inhibition [43,44]. From this perspective, the relatively reduced HR in the HBD group, in comparison with the LBD group (in the context of high emotional arousal), might indicate a greater negative reaction in the HBD group. A relevant finding here is the lack of habituation of SC levels in the HBD group. Blunted psychophysiological reactions have been associated with the presence of long-term chronic negative affective states [46]. This interpretation is in accordance with our self-reported data which show the existence of a negative affective state in HBD participants. However, other interpretations are also possible. For example, the results of HR and SC may reflect the fact that LBD participants focus more attention on their own self-perceived “beautiful” body parts and less on their “ugly” parts [23]. Furthermore, frequent body-checking behaviors could be related to increases in long-term anxiety that in turn may in part explain the absence of SC habituation in the HBD group [46].

Research on defensive behavior is also congruent with the above interpretation. Ortega-Roldán [47] found that BN patients displayed attenuated startle reflex amplitudes when viewing their own bodies in comparison with healthy controls. Given that the startle reflex is evoked when the organism has the intention of actively interacting with the environment [27], its inhibition in BN also supports the view of a passive-defensive coping style. This interpretation accords with our results and also suggests the presence of a passive-inhibited coping style in HBD women.

The results of the regression analysis between self-report and psychophysiological variables are in accordance with the above-mentioned dissociation effect, and with the presence of alterations in emotional regulation in HBD women. We have reported significant group differences in the relationship between self-reported emotional-cognitive and physiological indicators. Whereas in the LBD group VAS scores for *Ugly* and *Negative thoughts* were positively associated with HR, in the HBD group these associations were not significant and actually exhibited a trend towards a negative correlation. Similarly, sadness scores were positively associated with SC in the LBD group but no significant association was observed in HBD women. From a motivational point of view, in which emotions are considered as predispositions for action [27], associations are coherent and adaptive in the LBD group, in which they are associated with increased energetic mobilization in order to cope with potential threats. In the HBD group associations are incongruent from a motivational, adaptive, framework, and suggest again a passive-inhibited coping style predominance in the relationship of HBD women with their bodies.

Furthermore, these results suggest deficiencies in the generation of physiological correlates of emotions in relation to body dissatisfaction. In our HBD sample negative body image (e. g. *Ugly* feelings), emotions (e.g., sadness) and thoughts were not associated with greater physiological responses, and in some cases the trend in these associations (e.g., *Ugly*) actually runs contrary to a motivational perspective [27]. The strong association we found in the LBD group between *Ugly-Sadness* scores and HR-SC is noteworthy. These associations were observed both for the physiological averages through trials and for the specific responses obtained while participants were viewing their body parts. However, neither of these associations was observed in the HBD group.

Regarding possible limitations of our study, participants were encouraged to look at their different body parts via standardized audio-instructions, but we could not check whether they actually looked at these body parts. Additionally, as mentioned above, participants´ body checking frequency was not assessed. Another limitation concerns the fact that our baseline period, in light of our results, cannot be considered as a real baseline. Future studies should allow for the recording of a real baseline before the exposure trials, to prevent anticipation of such a stressful task. Furthermore, because our sample comprised university students, caution is required before generalizing the results to other female populations. Finally, we performed several correlation-regression analyses such that our data may be prone to alpha-error accumulation and random effects. Therefore, results concerning associations between self-report and psychophysiological variables should be regarded as exploratory; they require replication using a larger sample.

The clinical implications of our results should also be delineated. Firstly, other studies have demonstrated a positive association between previously-evoked autonomic arousal in response to a aversive stimuli-situation and the effectiveness of exposure therapies [28,29]. The blunted physiological reactivity we observed in the HBD group may represent a factor negatively impacting upon the effectiveness of mirror exposition techniques. Future studies should aim to specifically determine the relationship between previous physiological reactivity in response to viewing one’s own body in the mirror and the efficacy of mirror-exposure therapy. Secondly, our results suggest the need to modify the coping style of HBD women with respect to their body perception. Mirror therapy could be complemented by therapies aiming to change passive-avoidant strategies into more active ones. This could include reducing self-criticism, avoidance of social situations, behavioral inhibition, comparisons with others, social withdrawal, escape tendencies, ruminations, self-pity, resignation, substitutive satisfactions, etc., and greater exposure to social activities and relationships, positive auto-instructions and reinterpretations, and greater focus on the problem situation rather than the self, etc. This might be useful in reducing negative affect in such women, and perhaps also preventing the development of ED. Mirror exposure to one’s own image can be considered as a method by which to promote these alterations in coping, because the person is exposed to a previously avoided situation (i.e. observing her own body). Based on our results, we expect that clinical improvement using mirror exposure techniques in HBD women will be associated with an increase in physiological responses to body exposition and a normalization of associations between self-report and physiological variables.

In conclusion, HBD women experience large increases in negative emotions and cognitions when confronted with their own body in a mirror, but have blunted physiological reactivity in comparison to LBD women. In addition to this dissociation, the negative emotions and cognitions elicited by the mirror task in the HBD group were not associated with the generation of the appropriate physiological components of the emotion. Taken together, these findings suggest a passive-inhibited coping style in HBD women in the relationship they maintain with their body image. These alterations may be relevant in explaining the eating behavior regulation problems and emotional-body discomfort exhibited by these women.
